# Isometric and Isokinetic Assessment of Shoulder Rotator Muscle Strength, External/Internal Strength Ratios, and Fatigue Response in Competitive Male Gymnasts

**DOI:** 10.3390/sports14100433

**Published:** 2026-10-05

**Authors:** Dimitrios C. Milosis, Costas Dallas, Georgios Karamousalidis, Garifallia Daroglou, Evdoxia Kosmidou, Anatoli Petridou

**Affiliations:** 1 Laboratory of Evaluation of Human Biological Performance, Laboratory of Human Research and Sport Psychology, School of Physical Education and Sports Science, Department of Physical Education and Sport Sciences, Aristotle University of Thessaloniki, 57001 Thessaloniki, Greece; gkaramou@phed.auth.gr (G.K.); filio@phed.auth.gr (G.D.); ekosmido@phed.auth.gr (E.K.); apet@phed.auth.gr (A.P.); 2 School of Physical Education and Sport Science, Department of Physical Education and Sport Science, National and Kapodistrian University of Athens, 17237 Athens, Greece; kwnstantinos.dallas@gmail.com

**Keywords:** isokinetic dynamometry, peak torque, shoulder rotator strength, external/internal rotation strength ratios, muscle fatigability, artistic gymnastics

## Abstract

This study evaluated shoulder rotator muscle strength, conventional and functional external/internal rotation (ER/IR) strength ratios, and fatigue responses in competitive male artistic gymnasts, compared to a control group consisting of physical education (PE) students. Twenty-six participants were included: 13 competitive gymnasts (22.7 ± 2.3 years) and 13 PE students (22.6 ± 1.5 years), free of shoulder disorders. Shoulder rotator muscle strength was assessed isometrically and isokinetically (60°·s^−1^ and 180°·s^−1^) before and after a fatigue protocol comprising 50 maximal concentric internal and external rotations at 240°·s^−1^. Gymnasts demonstrated significantly higher body mass-normalized peak torque than PE students across the key strength conditions. Before fatigue, conventional and functional strength ratios were generally comparable between groups. Following fatigue, condition-specific changes in ER/IR strength ratios were observed, with gymnasts generally maintaining their pre-fatigue values. Normalized peak torque progressively declined across the 50-repetition protocol in both groups, with no significant between-group difference in the pattern or rate of torque decline. Exploratory analyses of derived fatigue-related measures showed limited and condition-specific between-group differences. Overall, competitive gymnasts demonstrated greater shoulder rotator strength capacity than PE students, whereas the findings did not support a generalized between-group difference in fatigue-related responses. Comprehensive assessment of shoulder strength, ER/IR strength ratios, and fatigue responses may provide useful information for performance monitoring and individualized training.

## 1. Introduction

Despite being one of the body’s most mobile yet inherently unstable joints, the shoulder is exposed to repetitive weight-bearing and externally applied forces during gymnastics skills such as tumbling, swinging, and dismounting [1,2,3,4,5]. These demands require gymnasts to develop high levels of upper-extremity strength while maintaining sufficient shoulder mobility to accommodate the large ranges of motion and demanding joint positions inherent to gymnastics skills [4,5]. In addition, effective neuromuscular control is required to generate and regulate force during these complex, high-demand movements [6,7,8,9]. Effective performance therefore depends on precise neuromuscular coordination and an appropriate relationship between internal and external rotator strength to support joint stability and control [5]. Unlike overhead throwing athletes, gymnasts predominantly perform closed kinetic chain activities with their hands supported on apparatuses or the floor, placing additional demands on upper-limb strength and joint stability [2].

Consequently, understanding shoulder function in gymnastics requires consideration not only of maximal rotator strength but also of the balance between external and internal rotator strength and the ability to sustain force production during repeated contractions. These characteristics can be assessed through peak torque (PT), conventional and functional external/internal rotation (ER/IR) strength ratios, referred to as the conventional ratio (CR) and functional ratio (FR), respectively, and changes in PT across repeated contractions [2,4,5]. Therefore, the assessment and development of gymnastics-specific shoulder strength, power, and coordination under both isometric and isokinetic conditions are essential for monitoring performance progression and supporting the execution of advanced gymnastics skills [7,8,9,10,11,12]. However, given that these high mechanical demands are repeatedly performed under fatigue-inducing conditions, neuromuscular fatigue represents a critical factor influencing both performance and injury risk.

Muscle fatigue is commonly defined as an acute, exercise-induced reduction in the ability to generate maximal voluntary muscle force or power. Beyond immediate performance impairment, neuromuscular fatigue can compromise neuromuscular control by increasing movement variability and reducing force steadiness [13,14]. Neuromuscular fatigue may also be associated with alterations in proprioception and postural stability, which may affect the ability to control joint position and movement during demanding tasks and may have implications for injury risk [15]. Accordingly, fatigue monitoring and management are increasingly prioritized as central components of performance optimization and injury prevention strategies [16].

Isokinetic dynamometry has been shown to provide reliable assessment of external and internal rotator strength, fatigability, and CR and FR [17,18], which are important indicators of shoulder function and stability [14,19,20,21,22,23,24]. This methodological framework has been widely applied in the literature; however, the current evidence base is characterized by a heterogeneous range of sports populations, limiting the generalizability of findings to specific sporting contexts [20]. Existing studies examining isokinetic characteristics of shoulder internal and external rotators have reported inconsistent findings regarding muscular strength, strength ratios, and fatigue-related measures across overhead athlete populations [21,25]. Furthermore, research efforts have predominantly focused on overhead sports, including swimming, volleyball, baseball, water polo, handball, rugby, and surfing [20,21,24,26,27], although swimming and volleyball remain the most extensively studied disciplines [27]. These limitations highlight the need for further research across sports with different upper-extremity loading demands to advance understanding of shoulder strength, muscle balance, and fatigability.

However, gymnastics remains comparatively under-investigated despite the substantial mechanical demands placed on the shoulder joint. In gymnastics specifically, only a limited number of studies have examined shoulder muscle performance [6,7,8,9,12,28,29], primarily focusing on movements such as shoulder protraction/retraction and flexion/extension, while, to our knowledge, no studies to date have investigated isometric and isokinetic (concentric and eccentric) strength, fatigue characteristics of the shoulder internal and external rotators, or related functional strength ratios in gymnastics. Such assessments are particularly relevant in gymnastics, where the shoulder complex is repeatedly exposed to high loading forces during weight-bearing, suspension, and swing activities. In men’s artistic gymnastics in particular, weight-bearing movements with the upper limbs, performed in either hanging or arm-support positions, place considerable demands on the shoulder region [4]. Adequate strength and an appropriate relationship between internal and external rotator strength are important for maintaining glenohumeral stability, controlling rotational forces, and sustaining performance during repetitive upper-extremity tasks. Furthermore, the evaluation of CR and FR, representing ER/IR strength ratios, may help identify neuromuscular adaptations or deficits associated with performance limitations and increased injury risk. Objective assessment of these parameters may therefore contribute to the development of sport-specific conditioning and injury prevention strategies for competitive gymnasts.

Therefore, the aims of this study were: (a) to compare maximal isometric and isokinetic strength of the shoulder internal and external rotator muscles between competitive male gymnasts and physically active physical education (PE) students; (b) to examine shoulder CR and FR before and after a fatigue protocol; and (c) to characterize fatigue responses, including the decline in normalized PT during a 50-repetition isokinetic protocol, between the two groups.

## 2. Materials and Methods

### 2.1. Study Design and Participants

A post hoc power analysis was conducted using G*Power Version (version 3.1.9.2, Kiel University, Kiel, Germany) for a repeated measures ANOVA (within–between interaction). With an alpha level of 0.05, a total sample size of 26 participants, two groups, and two measurements, the achieved statistical power was approximately 0.80 for detecting a medium effect size (f = 0.25), assuming a correlation of 0.50 among repeated measures and no sphericity correction of 1. A total of 26 male participants were recruited for this study, including 13 competitive gymnasts (age: 22.74 ± 2.34 years; body mass: 69.48 ± 4.58 kg; height: 168.81 ± 4.58; BMI: 24.39 ± 1.55) and 13 PE students (age: 22.62 ± 1.49 years; body mass: 75.09 ± 6.49 kg; height: 174.54 ± 4.07; BMI: 24.63 ± 1.76). All gymnasts were international-level athletes with >10 years of systematic training and a minimum training volume of 18 h·week^−1^. PE students were selected as the control group because they were physically active but non-competitive, with a substantial motor background and regular participation in practical sports activities through their university curriculum, involving at least 8 h of practical physical activity per week. This enabled them to complete the experimental and fatigue protocols while differing from gymnasts in long-term, sport-specific gymnastics training. Based on self-reported medical history, all participants were free from shoulder disorders that could have affected their performance during testing. The study was approved by the Research Ethics Committee of the School of Physical Education and Sport Science at Thessaloniki, Aristotle University of Thessaloniki (12 December 2023, approval number 166/2023), and all participants were fully informed about the purpose, procedures, and potential risks of the study. Written informed consent was obtained prior to participation, and all participants took part voluntarily.

### 2.2. Experimental Design

All laboratory assessments were conducted at the Laboratory of Evaluation of Human Biological Performance. All participants followed an identical testing protocol and were instructed to refrain from strenuous exercise for at least 24 h prior to testing. Following the assessment of height and body mass in minimal clothing using a Seca medical measurement station (Hamburg, Germany), participants completed a standardized warm-up consisting of: (a) 6 min on an arm-cycle ergometer (MONARK 881E; Monark Exercise AB, Vansbro, Sweden) with progressively increasing workload in both forward and backward directions (equally distributed), (b) 3 min of shoulder internal and external rotations using an elastic resistance band (2 sets of 15 repetitions), and (c) 3 min of passive static stretches targeting the internal and external rotator muscles [28].

**Figure 1:**
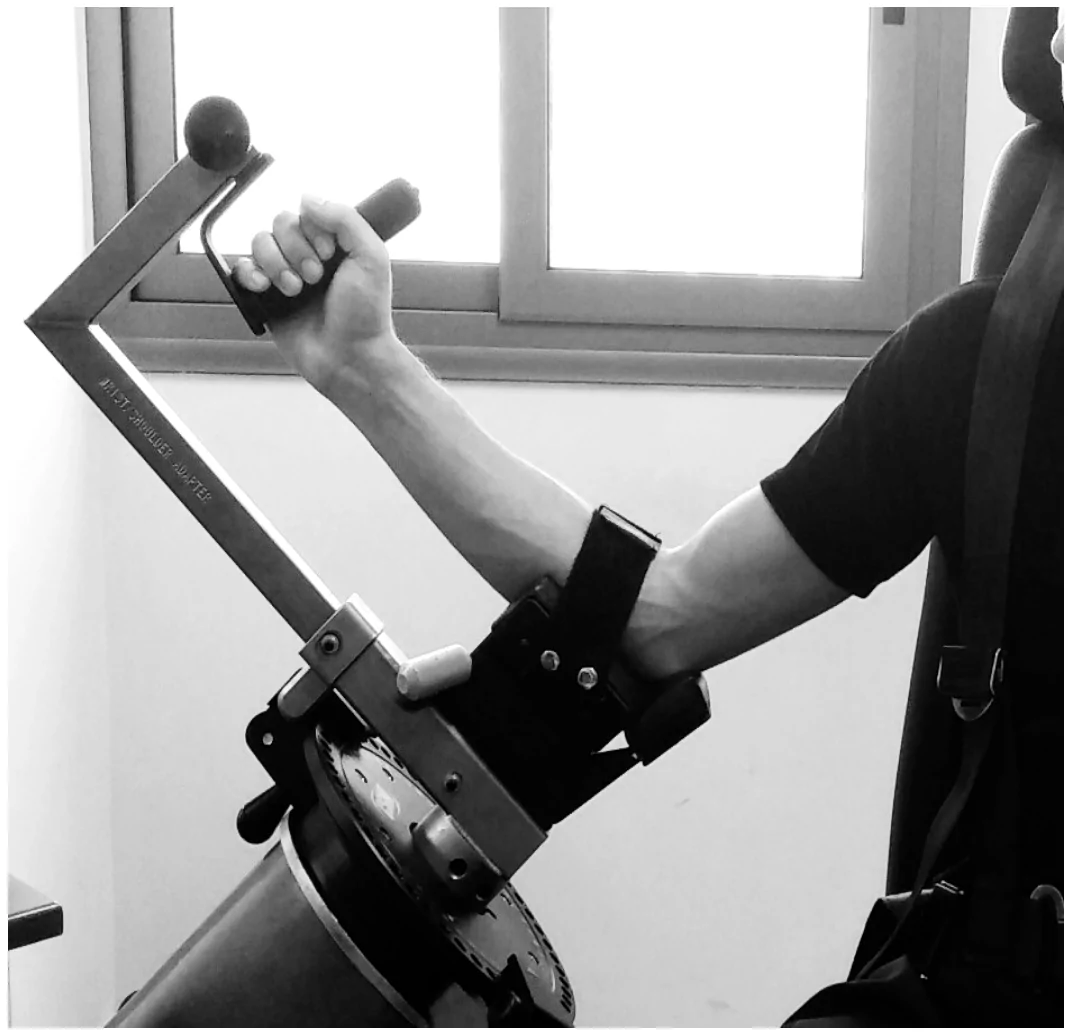
Experimental setup for seated shoulder internal/external rotation testing (Humac Norm 770).

Participants were then seated and securely stabilized on the dynamometer Humac Norm 770 (Computer Sports Medicine Inc./CSMi, Stoughton, MA, USA) using a safety belt. Researchers have shown that this dynamometer is reliable [17,18,28] and valid [7,8] in isokinetic muscle strength measurements of the shoulder. The tested arm was positioned in 45° abduction in the scapular plane, with the elbow flexed at 90°, and the forearm and wrist in neutral pronation/supination (Figure 1). The humerus was aligned with the rotational axis of the dynamometer. Evidence supports this position as the most reliable for IR and ER strength assessment [17,18]. Prior to testing, participants performed a specific warm-up on the dynamometer consisting of three submaximal concentric internal and external rotation repetitions at 60°·s^−1^ and five repetitions at 180°·s^−1^ to become familiar with the movement range and the accommodating resistance of the device.

For each arm, maximal voluntary isometric contraction (MVIC) of the shoulder internal and external rotators was assessed at 0° of shoulder rotation (neutral position) using two 5 s maximal contractions. Participants were instructed to exert maximal effort throughout each trial (as fast and forceful as possible), and the highest PT obtained was used for analysis. The isokinetic assessment included three maximal concentric contractions at 60°·s^−1^, four at 180°·s^−1^, and three maximal eccentric contractions at 60°·s^−1^ for both internal and external shoulder rotation, performed before and after the fatigue protocol. The assessments were performed in the following order: isometric contractions, concentric contractions at 60°·s^−1^, concentric contractions at 180°·s^−1^, and eccentric contractions at 60°·s^−1^. The same sequence was used for all participants to standardize the testing procedure and minimize potential carry-over effects between testing conditions. The fatigue protocol was performed separately for each arm and consisted of 50 consecutive repetitions of maximal concentric internal and external shoulder rotations at an angular velocity of 240°·s^−1^, in accordance with the protocol described by Gaudet et al. [30].

Participants were instructed to maintain an upright posture throughout testing to minimize trunk involvement in torque production. A 30 s passive recovery period was provided between successive isometric contractions in the same direction. A 60 s passive recovery period was provided when changing the direction of contraction and between different contraction modes (isometric, concentric, and eccentric) to minimize potential fatigue effects on subsequent assessments. Such rest intervals have been considered sufficient for the recovery and reliable reassessment of muscle performance [31]. Verbal encouragement was provided throughout the fatigue protocol to ensure maximal effort. Gravity correction was performed using the dynamometer’s software (Humac2015) [32].

### 2.3. Statistical Analysis

Statistical analyses were performed using IBM SPSS Statistics (Version 29, IBM Corp., Armonk, NY, USA). Strength outcomes are presented as mean ± standard error (SE), whereas fatigue-related indices are presented as mean ± standard deviation (SD). Normality of the variables was assessed using the Shapiro–Wilk test, while homogeneity of variances between groups was examined using Levene’s test for equality of variances. The equality of covariance matrices was evaluated using Box’s M test. Sphericity was assessed with Mauchly’s test, and Greenhouse–Geisser corrections were applied when necessary.

Given the sample size (*n* = 13 per group) and the potential for type I error inflation associated with multiple comparisons, a hypothesis-driven analytical strategy was adopted, with outcomes classified a priori as key confirmatory or exploratory measures. Key inferential outcomes were defined for the dominant shoulder and included body mass-normalized PT during isometric contractions at 0°, concentric contractions at 60°·s^−1^, and eccentric contractions at 60°·s^−1^ for both internal and external rotation, as well as conventional ER/IR strength ratios at 0° and 60°·s^−1^ and the functional ER/IR ratio at 60°·s^−1^. The remaining strength and ratio conditions, including all non-dominant shoulder measures and assessments at 180°·s^−1^, were considered exploratory and were interpreted primarily descriptively. For the fatigue protocol, the primary inferential analysis was the Group × Block interaction for normalized PT across the 10 repetition blocks, whereas fatigue index, absolute torque drop, and fatigue slope were considered exploratory secondary measures and were interpreted primarily using effect sizes, confidence intervals, and descriptive patterns.

For the key outcomes, separate two-way mixed ANOVAs were conducted, with Group (gymnasts vs. PE students) as the between-subjects factor and Time (pre vs. post) as the within-subject factor. Significant Group × Time interactions were followed by simple-effects analyses, with Bonferroni-adjusted pairwise comparisons used to examine between-group differences at each time point and pre–post changes within each group. Peak torque was defined as the maximum torque obtained under each testing condition and was normalized to body mass (values expressed as Nm·kg^−1^ × 100). Exploratory PT and ratio outcomes were retained to provide a comprehensive characterization of shoulder function but were summarized descriptively and interpreted as hypothesis-generating.

The CR was calculated as follows: (a) isometric ER at 0° divided by isometric IR at the same angle, multiplied by 100; (b) concentric ER at 60°·s^−1^ and 180°·s^−1^ divided by concentric IR at the corresponding angular velocities, multiplied by 100; and (c) eccentric ER at 60°·s^−1^ divided by eccentric IR at the same angular velocity, multiplied by 100. The FR was calculated as eccentric ER at 60°·s^−1^ divided by concentric IR at 60°·s^−1^, multiplied by 100.

For the key fatigue analysis, normalized PT values for dominant internal and external rotation were averaged into 10 consecutive blocks comprising five repetitions each. A mixed-design repeated-measures ANOVA was performed with Block (1–10) as the within-subject factor and Group (gymnasts vs. PE students) as the between-subject factor. The Group × Block interaction was considered the primary inferential test for evaluating whether the pattern of torque decline differed between groups. The corresponding non-dominant shoulder analyses were considered exploratory and were interpreted descriptively.

Given the sample size and the number of assessed outcomes, additional fatigue-related measures were treated as exploratory. These included fatigue slope, fatigue index, and absolute torque drop. They were retained to provide complementary information on the fatigue response but were not considered confirmatory hypothesis tests. Their interpretation therefore emphasized group differences, effect sizes, and 95% confidence intervals rather than statistical significance alone. Fatigue slope was calculated from the linear regression equation fitted to the decline in normalized PT (%MVIC) across the 10 consecutive blocks of five repetitions during the fatigue protocol. The regression coefficient (slope) represented the rate of decline in torque production and was expressed as %MVIC per block. Fatigue index was calculated as [(initial normalized PT − final normalized PT)/initial normalized PT] × 100 and expressed as a percentage. Absolute torque drop was calculated as the difference between initial and final normalized PT values and expressed as %MVIC. Statistical significance for the key inferential analyses was set at *p* < 0.05. Effect sizes were reported as partial eta squared (η*p*^2^) for ANOVA results and Cohen’s d for between-group comparisons, together with 95% confidence intervals where appropriate. Exploratory outcomes were not used to support confirmatory between-group claims and were interpreted primarily on the basis of effect magnitude, direction, and precision.

## 3. Results

Assumptions were assessed using the Shapiro–Wilk, Levene’s, and Box’s M tests, with no significant violations observed. Sphericity was tested with Mauchly’s test, and Greenhouse–Geisser corrections were applied where appropriate.

### 3.1. Between-Group Differences in Peak Torque and ER/IR Strength Ratios Before and After the Fatigue Protocol

Gymnasts generally demonstrated higher body-mass-normalized PT than PE students across the assessed strength conditions. Significant main effects of Group were observed for several isometric and isokinetic PT measures, with the magnitude and direction of the between-group differences varying according to rotation direction, contraction mode, angular velocity, and shoulder. Among the key outcomes, a significant Group × Time interaction was observed for external isometric PT at 0° in the dominant shoulder, indicating a condition-specific difference in the change in PT from pre- to post-fatigue. Significant main effects of Time were observed for several PT measures, indicating reductions in torque following the fatigue protocol. Detailed descriptive statistics, effect sizes, confidence intervals, and inferential statistics are presented in Table 1.

No significant between-group differences were observed in the key CR and FR outcomes before the fatigue protocol. Following fatigue, no significant between-group differences were observed in the key ratio outcomes. Full descriptive and inferential statistics are provided in Table 2. Exploratory analyses of the remaining strength and ratio conditions—including assessments of the non-dominant shoulder and evaluations at 180°·s^−1^—are provided in Supplementary Tables S1 and S2 and Figures S1--S4, with their statistical outcomes reported alongside descriptive measures.

### 3.2. Fatigue Response During the 50-Repetition Protocol

Normalized PT progressively declined across the 50-repetition fatigue protocol in both groups, as demonstrated by significant main effects of Block for all rotation conditions (all *p* < 0.001). No significant Group × Block interactions were observed, indicating that the pattern of torque decline across the 10 blocks did not differ significantly between gymnasts and PE students (Table 3).

Exploratory analysis of the derived fatigue-related measures showed a lower fatigue index in gymnasts during dominant internal rotation, whereas no consistent between-group pattern was observed for fatigue index, absolute torque drop, or fatigue slope across the remaining conditions (Table 4). These findings were considered exploratory and should therefore be interpreted cautiously given the number of comparisons performed.

**Figure 2:**
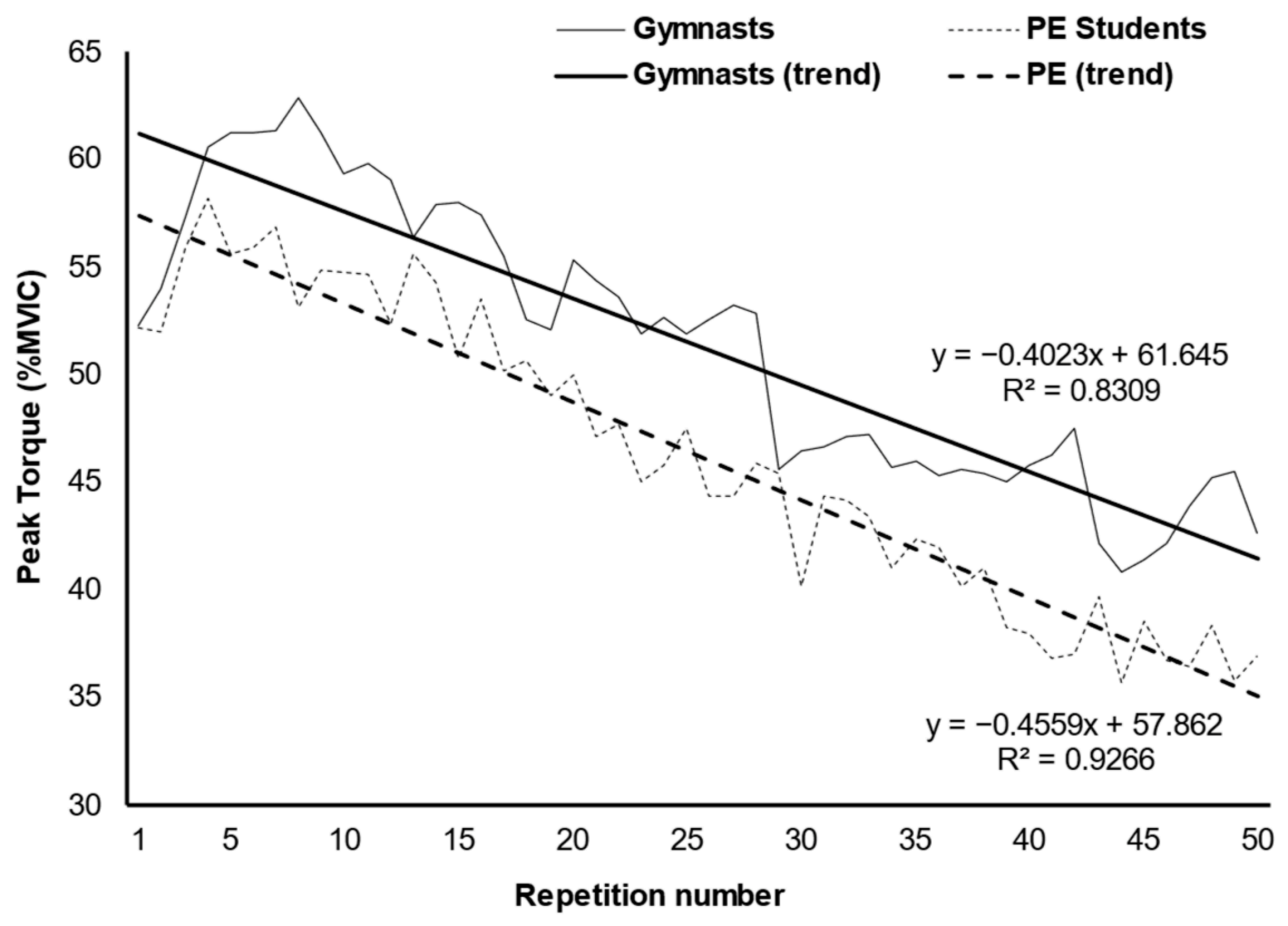
Linear regression analysis of dominant shoulder isokinetic concentric internal rotation PT (expressed as %MVIC at 0°, thereby accounting for differences in maximal strength between groups) across a 50-repetition fatigue protocol (240°·s^−1^) in gymnasts (solid line) and physical education students (dashed line). The slope of each regression line represents fatigue rate, while R^2^ indicates goodness of fit.

**Figure 3:**
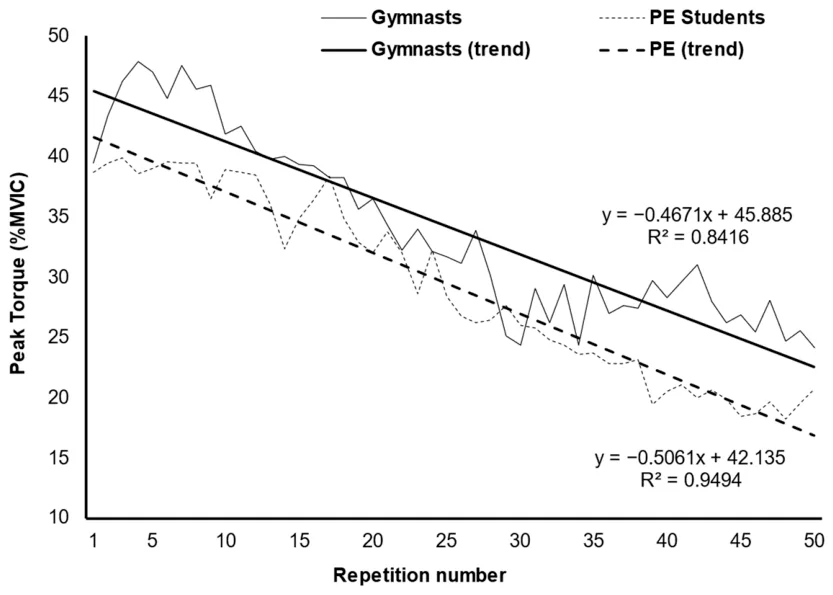
Linear regression analysis of dominant shoulder isokinetic concentric external rotation PT (expressed as %MVIC at 0°, thereby accounting for differences in maximal strength between groups) across a 50-repetition fatigue protocol (240°·s^−1^) in gymnasts (solid line) and physical education students (dashed line). The slope of each regression line represents the fatigue rate, while R^2^ indicates goodness of fit.

The group mean torque curves showed a progressive decline in normalized PT across the 50 repetitions in both groups (Figure 2 and Figure 3). Gymnasts generally maintained higher normalized torque values throughout the protocol, consistent with their higher baseline strength; however, the absence of significant Group × Block interactions does not provide evidence of a between-group difference in the rate of torque decline. Exploratory analyses of the remaining fatigue conditions are provided in Supplementary Tables S3 and S4 and Figures S5 and S6 and are interpreted descriptively.

## 4. Discussion

### 4.1. Shoulder Rotator Muscle Strength

Overall, gymnasts demonstrated greater PT than PE students across most strength variables, consistent with the high upper-body strength demands of competitive gymnastics [2,4,5,6,7,8,9,10]. Gymnastics requires repeated production and control of force during weight-bearing, suspension, swinging, and other apparatus-specific skills, placing substantial mechanical demands on the shoulder complex. The greater strength capacity observed in gymnasts may therefore be consistent with long-term exposure to these sport-specific demands. However, given the cross-sectional design, these findings should not be interpreted as direct evidence of training-induced adaptations.

The association between shoulder strength and shoulder pathology has been investigated extensively in overhead and upper-extremity sports, including volleyball, baseball, handball, rugby, swimming, surfing, and water polo [20,21,24,26,27]. However, strength capacity alone may not fully account for shoulder pathology in gymnastics. In their study, Gendre and Boileau [4] reported that, in three shoulders, injury occurred during strength-and-hold positions on the rings and was attributed to isometric muscle contraction against gravity. Although based on a limited number of cases, these findings suggest that shoulder pathology may occur even during apparatus-specific tasks requiring exceptionally high levels of shoulder strength, indicating that force-producing capacity alone may not be sufficient to protect against injury. While these findings provide a theoretical framework, they must be interpreted with caution due to the cross-sectional design and limited sample size.

In the same study, Gendre and Boileau [4] reported that in 27 of 30 shoulders (90%), the mechanism of injury involved traction in forced flexion–rotation during suspension-based skills performed with “locked hands” on parallel bars, horizontal bar, or rings. Injuries were sustained most frequently on the rings, followed by the horizontal bar and parallel bars, and 17 shoulders presented either a history of instability or instability-related lesions. Based on the available literature, the authors further suggested that shoulder instability in the absence of prior dislocation or subluxation may result from progressive capsular stretching due to repetitive overload and/or muscle imbalance in overhead athletes. This mechanism appears highly relevant to artistic gymnastics, where the shoulder is repeatedly exposed to traction and extreme loading demands. In this context, the assessment of shoulder CR and FR becomes particularly important, as altered agonist–antagonist balance might theoretically be associated with compromised dynamic glenohumeral stability, highlighting a potential factor that warrants further investigation regarding shoulder health in gymnastics.

### 4.2. Conventional and Functional Strength Ratios

The balance between shoulder IR and ER strength is an important parameter in the interpretation of isokinetic testing. In healthy individuals, CR has been reported to approximate 66%, with values ranging from approximately 51% to 80% depending on testing velocity, population, and sporting discipline [22,23,26,27,33,34,35,36]. Functional ratios, calculated as eccentric ER torque relative to concentric IR torque, have also been proposed as clinically relevant indicators of dynamic shoulder stability. These ratios often approach or exceed 1.0, reflecting the substantial eccentric demands placed on the ER during deceleration, although lower values have been reported in sports such as handball and tennis, highlighting sport-specific adaptations [14,22,26,27,36,37,38].

This is clinically relevant because inadequate ER strength relative to the IR may compromise shoulder deceleration and dynamic stability during overhead movements, increasing mechanical stress on the glenohumeral joint [39]. Moreover, ER fatigue may produce functional alterations similar to those associated with muscular imbalance, potentially contributing to glenohumeral instability and subacromial impingement [40].

In the present study, CR ranged from 29.51% to 47.42%, whereas FR ranged from 45.71% to 56.10%. The comparatively lower CR than those commonly reported may reflect methodological differences in testing protocols, angular velocities, contraction modes, and participant characteristics, as well as sport-specific adaptations. Therefore, direct comparisons across studies should be interpreted cautiously given the heterogeneity of isokinetic assessment protocols and athletic populations.

No significant between-group differences were observed in either ratio. However, a different pattern emerged following the fatigue protocol: gymnasts generally maintained or slightly increased both CR and FR, whereas PE students tended to show reduced ratios, particularly in the dominant shoulder. This pattern was more evident for the FR. Although these findings should be interpreted cautiously, they may be consistent with sport-specific adaptations associated with long-term gymnastics training. The maintenance of the ratios in gymnasts may indicate a more stable relationship between IR and ER torque during repeated contractions and potentially maintain dynamic glenohumeral stability during repetitive high-load tasks.

Although no formal apparatus-specific analyses were performed, individual examination suggested that PB and HB specialists tended to exhibit lower torque production, reduced fatigue resistance, lower strength ratios, and greater side-to-side asymmetry. These observations are exploratory given the limited sample and descriptive nature of the analysis. The high mobility, extreme shoulder positions, and unilateral loading demands associated with apparatus-specific skills may contribute to individual differences in shoulder strength and rotational strength relationships [4,41]. Further research using larger samples and apparatus-specific analyses is required to determine whether such patterns represent meaningful specialization-related characteristics.

### 4.3. Fatigability of the Shoulder Rotator Muscles

To account for differences in maximal strength capacity, torque values were normalized to MVIC, allowing fatigability to be assessed independently of absolute strength. Fatigability was further characterized by the decline in normalized PT throughout the 50-repetition protocol and by derived fatigue-related indices, including fatigue index, absolute torque drop, and fatigue slope [27,30,42,43]. Beyond reductions in force production, neuromuscular fatigue may impair force control and movement consistency by increasing variability and reducing force steadiness [13,14], potentially compromising sensorimotor control and dynamic shoulder stability in overhead athletes [15]. Accordingly, assessment of fatigability provides information complementary to maximal strength and may be relevant to performance monitoring and to strategies aimed at maintaining shoulder function and potentially reducing fatigue-related demands on the shoulder during repeated tasks [15,22,27,30,43].

Overall, both groups demonstrated a progressive decline in normalized torque throughout the 50-repetition protocol, indicating substantial fatigability of the shoulder internal and external rotator muscles. This observation was supported by the significant main effect of repetition block identified in most testing conditions (all *p* < 0.001). However, no significant Group × Block interactions were observed, indicating that the pattern of torque decline across the protocol did not differ significantly between gymnasts and PE students. The main effect of Group was also not significant. Thus, although gymnasts generally maintained higher normalized torque values throughout the protocol, consistent with their greater baseline strength capacity, this finding should not be interpreted as evidence of a different rate of fatigue.

The derived fatigue-related measures provided limited evidence of between-group differences. A significant difference in fatigue index was observed only during dominant internal rotation (*p* = 0.025), with lower values in gymnasts, whereas the between-group difference in absolute torque drop did not reach statistical significance (*p* = 0.064). Given the number of fatigue-related comparisons, the isolated finding for dominant internal rotation should be interpreted cautiously and should not be considered evidence of a generalized difference in fatigability between groups.

The relatively limited between-group differences may partly reflect the characteristics of the comparison group. Although the PE students were not competitive athletes, they were physically active and had substantial exposure to a wide range of sports and practical training as part of their university curriculum. This relatively broad physical activity background may have contributed to their ability to sustain repeated maximal contractions and reduced the magnitude of between-group differences. Conversely, the specific demands of competitive gymnastics, characterized by repetitive high-intensity loading and sustained and repeated muscle contractions, may contribute to the ability to maintain force production during repeated contractions. However, this interpretation remains speculative, and the absence of significant Group × Block interactions does not support a generalized difference in fatigue resistance between the groups.

Finally, the fitted linear trendlines indicated that the decline in normalized torque across the 50 repetitions followed an approximately linear pattern in both groups, providing a useful descriptive representation of torque decline during repeated maximal contractions. However, these trendlines are descriptive and do not provide evidence of a between-group difference in the rate of fatigue, which was not supported by the inferential analysis.

### 4.4. Limitations

Several limitations of the present study should be acknowledged. First, the cross-sectional design precludes conclusions regarding the predictive value of shoulder strength characteristics and fatigability for future shoulder injuries or pathology. Second, the study included only adult male competitive gymnasts; consequently, the findings cannot be generalized to female gymnasts, younger athletes, or other competitive levels. Third, the gymnast cohort was not entirely homogeneous with respect to apparatus specialization. Although all participants were highly trained, they specialized in different apparatus, which may have influenced shoulder strength characteristics and fatigue responses. Fourth, although the comparison group did not consist of competitive athletes, the PE students had a substantial motor background and maintained a relatively high level of physical fitness through regular participation in practical sports activities as part of their university curriculum. This may have reduced the magnitude of between-group differences. Finally, the fatigue protocol was limited to repeated isokinetic shoulder internal and external rotations, and no neuromuscular measures, such as surface electromyography, were collected. Consequently, the physiological mechanisms underlying the observed fatigue responses could not be directly examined. In addition, the relatively small sample size may have limited the statistical power to detect between-group differences in some fatigue-related outcomes, particularly those with smaller effect sizes.

Future longitudinal studies are needed to clarify whether the neuromuscular characteristics observed in artistic gymnasts, such as maximal torque production, fatigue-related responses, conventional and functional strength ratios, and external/internal rotator strength balance, are associated with long-term training exposure and subsequent shoulder health. Moreover, the establishment of a comprehensive database of shoulder neuromuscular profiles across different ages, competitive levels, and apparatus specializations would provide valuable reference values for researchers and practitioners, facilitating the identification of atypical strength profiles and the development of individualized training and rehabilitation strategies. Future investigations should also incorporate neuromuscular assessment techniques, such as surface electromyography, to further explore muscle activation patterns, coordination strategies, and fatigue-related alterations during shoulder-specific tasks.

## 5. Conclusions

Competitive male gymnasts demonstrated greater body mass-normalized shoulder rotator PT than physically active PE students across most testing conditions, whereas strength ratios were generally comparable between groups. Peak torque declined progressively during the 50-repetition fatigue protocol in both groups, with no significant between-group differences in the pattern of torque decline. Although a between-group difference was observed for the fatigue index during dominant internal rotation, this isolated finding should be interpreted cautiously in the context of the multiple comparisons performed. Overall, long-term artistic gymnastics participation appears to be associated with greater shoulder rotator capacity, but the present findings do not support a generalized difference in fatigability between groups. Comprehensive assessment of shoulder strength and fatigability may support performance monitoring and individualized training, while normative databases across age, competitive level, and apparatus specialization could further improve athlete-specific interpretation and training and rehabilitation strategies.

## Figures and Tables

**Table 1:** Body mass-normalized PT values (M ± SE) before and after the fatigue protocol and mixed ANOVA results for the dominant shoulder.

	Gymnasts Pre	PE Pre	Gymnasts Post	PE Post									
Variable	*M* ± *SE*	*M* ± *SE*	*M* ± *SE*	*M* ± *SE*	Group *F*	*p*	η*p*^2^	Time *F*	*p*	η*p*^2^	Interaction *F*	*p*	η*p*^2^
Isom IR 0°	158 ± 6	116 ± 6	143 ± 6	95 ± 6	36.42	<0.001	0.60	50.83	<0.001	0.68	1.32	0.263	0.05
Isom ER 0°	61 ± 3	45 ± 3	54 ± 3	31 ± 3	30.31	<0.001	0.59	54.52	<0.001	0.69	4.38	0.047	0.15
Con IR 60°·s^−1^	128 ± 5	94 ± 5	122 ± 5	86 ± 5	24.89	<0.001	0.51	17.84	<0.001	0.43	0.23	0.637	0.01
Con ER 60°·s^−1^	49 ± 3	39 ± 3	47 ± 3	32 ± 3	10.85	0.003	0.31	12.99	<0.001	0.35	4.14	0.053	0.15
Ecc IR 60°·s^−1^	154 ± 7	115 ± 7	142 ± 6	104 ± 6	20.44	<0.001	0.46	12.03	0.002	0.33	0.18	0.895	0.00
Ecc ER 60°·s^−1^	64 ± 4	52 ± 4	68 ± 6	47 ± 6	9.15	0.006	0.28	0.05	0.824	0.00	1.28	0.269	0.05

Note. All torque values are expressed as Nm·kg^−1^ × 100. Abbreviations: *M*, mean; *SE*, standard error; *F*, F statistic; *p*, probability value; η*p*^2^, partial eta squared; Isom, isometric contraction; IR, internal rotation; ER, external rotation; Con, concentric contraction; Ecc, eccentric contraction.

**Table 2:** Conventional and functional external/internal ratios (M ± SE) before and after the fatigue protocol and mixed ANOVA results for the dominant shoulder.

	Gymnasts Pre	PE Pre	Gymnasts Post	PE Post									
Variable	*M* ± *SE*	*M* ± *SE*	*M* ± *SE*	*M* ± *SE*	Group *F*	*p*	η*p*^2^	Time *F*	*p*	η*p*^2^	Interaction *F*	*p*	η*p*^2^
Isom ER 0°/Isom IR 0°	38 ± 2	39 ± 2	38 ± 2	34 ± 2	0.68	0.417	0.03	6.49	0.018	0.21	2.98	0.097	0.11
Con ER/Con IR 60°·s^−1^	39 ± 3	42 ± 3	39 ± 2	37 ± 2	0.01	0.931	0.00	5.87	0.023	0.20	7.33	0.012	0.23
Ecc ER/Ecc IR 60°·s^−1^	45 ± 2	45 ± 2	46 ± 3	43 ± 3	0.40	0.534	0.02	0.10	0.761	0.00	0.42	0.521	0.02
Ecc ER/Con IR 60°·s^−1^	51 ± 3	56 ± 3	56 ± 5	55 ± 5	0.19	0.669	0.01	0.32	0.578	0.01	0.55	0.465	0.02

Abbreviations: *M*, mean; *SE*, standard error; *F*, F statistic; *p*, probability value; η*p*^2^, partial eta squared; Isom, isometric contraction; IR, internal rotation; ER, external rotation; Con, concentric contraction; Ecc, eccentric contraction; Ecc ER/Con IR, functional ratio.

**Table 3:** Mixed repeated-measures ANOVA results for normalised peak torque across the 50-repetition fatigue protocol.

Variable	Group *F*	*p*	η*p*^2^	Block *F*	*p*	η*p*^2^	Interaction *F*	*p*	η*p*^2^
IR Dom	2.95	0.099	0.11	25.69	<0.001	0.94	0.83	0.492	0.03
ER Dom	3.97	0.058	0.14	17.95	<0.001	0.91	1.54	0.223	0.06

Note: Greenhouse–Geisser corrected values were used where the assumption of sphericity was violated. Abbreviations: *F*, F statistic; *p*, probability value; η*p^2^*, partial eta squared; Block, 10 consecutive blocks of 5 repetitions; IR Dom, internal rotation for the dominant side; ER Dom, external rotation for the dominant side.

**Table 4:** Exploratory descriptive comparison of fatigue-related indices between groups.

		Group				
Rotation Condition	Variable	Gymnasts Mean ± SD	PE Students Mean ± SD	*t*	95%CI	Cohen’s d
IR Dom	Fatigue index	22 ± 12.51	33.00 ± 9.82	−2.40	[−19.68, 1.47]	−0.94
	Absolute drop	13.03 ± 7.14	18.10 ± 6.16	−1.94	[−10.47, 0.33]	−0.76
	Fatigue slope	−0.40 ± 0.14	−0.46 ± 0.20	0.88	[−0.08, 0.20]	0.33
ER Dom	Fatigue index	41.20 ± 16.26	47.55 ± 17.20	−0.97	[−17.90, 7.20]	−0.38
	Absolute drop	19.57 ± 10.84	18.64 ± 7.56	0.25	[−6.64, 8.49]	0.10
	Fatigue slope	−0.47 ± 0.25	−0.48 ± 0.19	0.12	[−0.17, 0.19]	0.05

Note. Values are presented as Mean ± SD. The *p*-values and 95% CIs were derived from a two-sided independent samples *t*-test. Cohen’s d was calculated using the pooled standard deviation; negative values indicate lower values in gymnasts than in PE students. Abbreviations: SD, Standard Deviation; CI, Confidence Interval; IR Dom, internal rotation for the dominant side; ER Dom, external rotation for the dominant side.

## Data Availability

The data that support the findings of this study are openly available in the repository of the Hellenic Academic Research Data Management Initiative at DOI: 10.26255/heal.3x5f-pbmp.

## Supplementary Materials

The following supporting information can be downloaded at: https://www.mdpi.com/article/10.3390/sports14100433/s1, Figure S1. Exploratory normalized peak torque (PT) of dominant shoulder for gymnasts and PE students during isometric, concentric, and eccentric contractions at different angular velocities, before and after the fatigue protocol. Figure S2. Exploratory normalized peak torque (PT) of non-dominant shoulder for gymnasts and PE students during isometric, concentric, and eccentric contractions at different angular velocities, before and after the fatigue protocol. Figure S3. Exploratory conventional and functional external/internal ratios of dominant shoulder for gymnasts and PE students, before and after the fatigue protocol. Data are expressed as means ± standard errors. Figure S4. Exploratory conventional and functional external/internal ratios of non-dominant shoulder for gymnasts and PE students, before and after the fatigue protocol. Figure S5. Exploratory linear regression analysis of non-dominant shoulder isokinetic concentric internal rotation peak torque (expressed as %MVIC at 0°, thereby accounting for differences in maximal strength between groups) across a 50-repetition fatigue protocol (240°·s^−1^) in gymnasts (solid line) and physical education students (dashed line). The slope of each regression line represents fatigue rate, while R^2^ indicates goodness of fit. Figure S6. Exploratory linear regression analysis of non-dominant shoulder isokinetic concentric external rotation peak torque (expressed as %MVIC at 0°, thereby accounting for differences in maximal strength between groups) across a 50-repetition fatigue protocol (240°·s^−1^) in gymnasts (solid line) and physical education students (dashed line). The slope of each regression line represents fatigue rate, while R^2^ indicates goodness of fit. Table S1. Exploratory body mass-normalized peak torque (PT) values (M ± SE) before and after the fatigue protocol and mixed ANOVA results for the non-dominant shoulder. Table S2. Exploratory conventional and functional external/internal ratios (M ± SE) before and after the fatigue protocol and mixed ANOVA results for the non-dominant shoulder. Table S3. Exploratory mixed repeated-measures ANOVA results for normalised peak torque across the 50-repetition fatigue protocol. Table S4. Exploratory descriptive comparison of fatigue-related indices between groups.

## Abbreviations

The following abbreviations are used in this manuscript:

PE	Physical education
ER/IR	External/internal rotation
CR	Conventional strength ratios
FR	Functional strength ratios
MVIC	Maximal voluntary isometric contraction
PT	Peak torque
η*p*^2^	Partial eta squared
