# Supplementary material for: Isometric and Isokinetic Assessment of Shoulder Rotator Muscle Strength, External/Internal Strength Ratios, and Fatigue Response in Competitive Male Gymnasts
Source: Sports (Basel). 2026 Oct 5;14(10):433. doi: 10.3390/sports14100433 (PMC13709291; doi:10.3390/sports14100433)
Supplement: Supplementary file 1 [file sports-14-00433-s001.zip › sports-4540623-supplementary.docx.author proof.pdf]

sports

Supplementary Material

Additional descriptive and exploratory findings, including between-group differences in peak torque, strength ratios, and linear regression analyses of the non-dominant shoulder across the 50-repetition protocol (%MVIC), are provided in Supplementary Figures S1-S6.

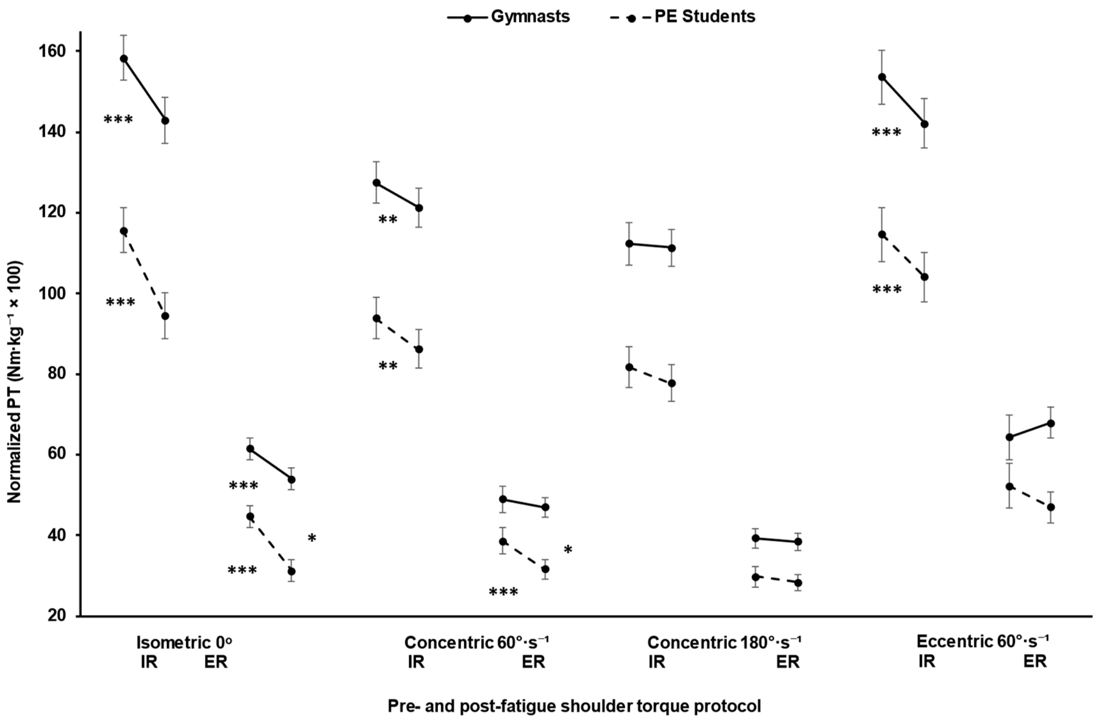

Figure S1. *Exploratory normalized peak torque (PT) of dominant shoulder for gymnasts and PE students during isometric, concentric, and eccentric contractions at different angular velocities, before and after the fatigue protocol. Data are expressed as means ± standard errors. * $p < 0.05$, ** $p < 0.01$, and *** $p < 0.001$ denote significant within-group differences from the corresponding pre-fatigue values.*

Note: IR, internal rotation; ER, external rotation.

 https://doi.org/10.3390/sports14100433

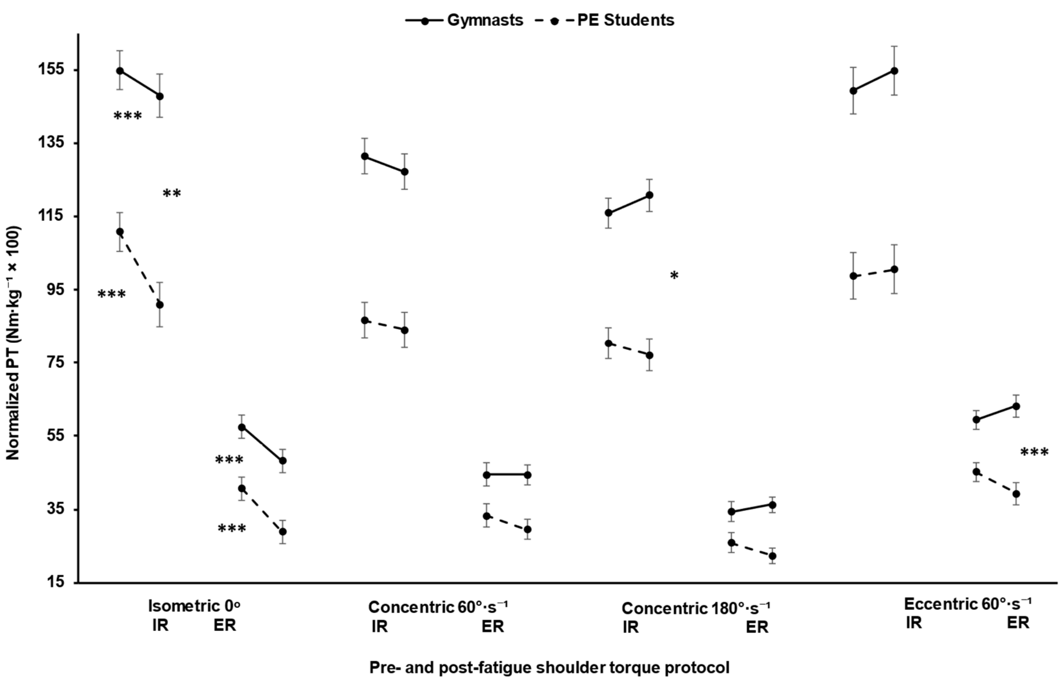

~~***Figure* S2. Exploratory normalized peak torque (PT) of non-dominant shoulder for gymnasts and PE students during**~~ **isometric**, *concentric*, and eccentric contractions at different angular velocities, before and after the fatigue protocol. Data are expressed as means ± standard errors. ∗ $p < 0.05$, ∗∗ $p < 0.01$, and ∗∗∗ $p < 0.001$ denote significant within-group differences from the corresponding pre-fatigue values.

Note: IR, internal rotation; ER, external rotation.

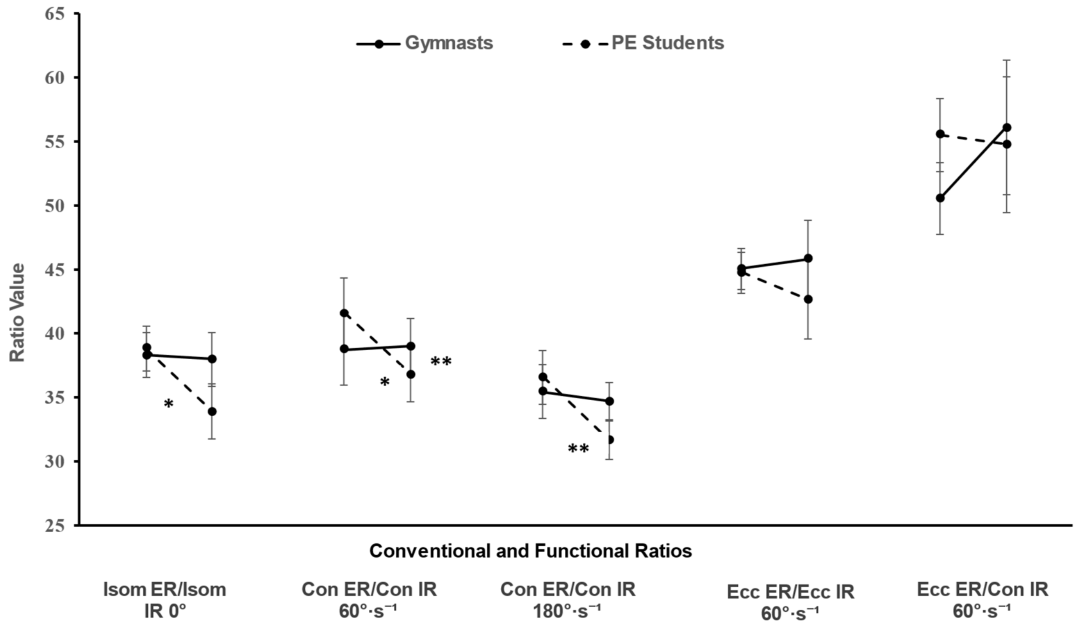

Figure S3. Exploratory conventional and functional external/internal ratios of dominant shoulder for gymnasts and PE students, before and after the fatigue protocol. Data are expressed as means ± standard errors.

∗ $p < 0.05$ and ∗∗ $p < 0.01$ denote significant differences from pre-fatigue values.

Note: IR, internal rotation; ER, external rotation.

https://doi.org/10.3390/sports14100433

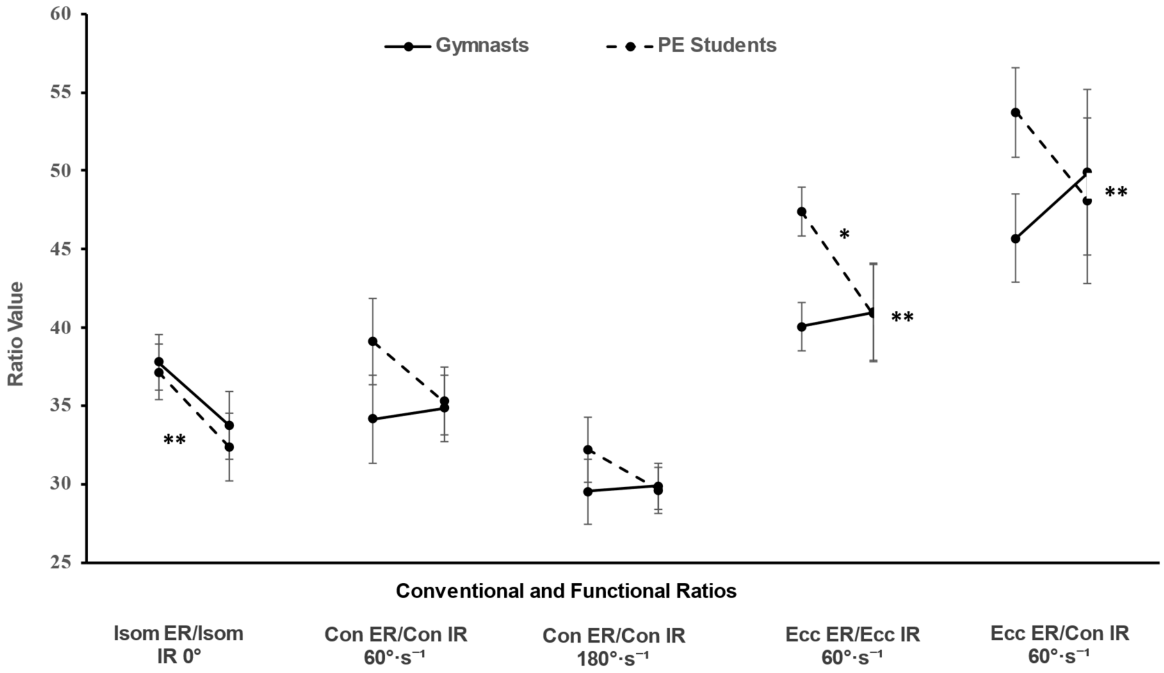

Figure S4. Exploratory conventional and functional external/internal ratios of non-dominant shoulder for gymnasts and PE students, before and after the fatigue protocol. Data are expressed as means ± standard errors.

∗ $p < .05$ and ∗∗ $p < .01$ denote significant differences from pre-fatigue values.

Note: IR, internal rotation; ER, external rotation.

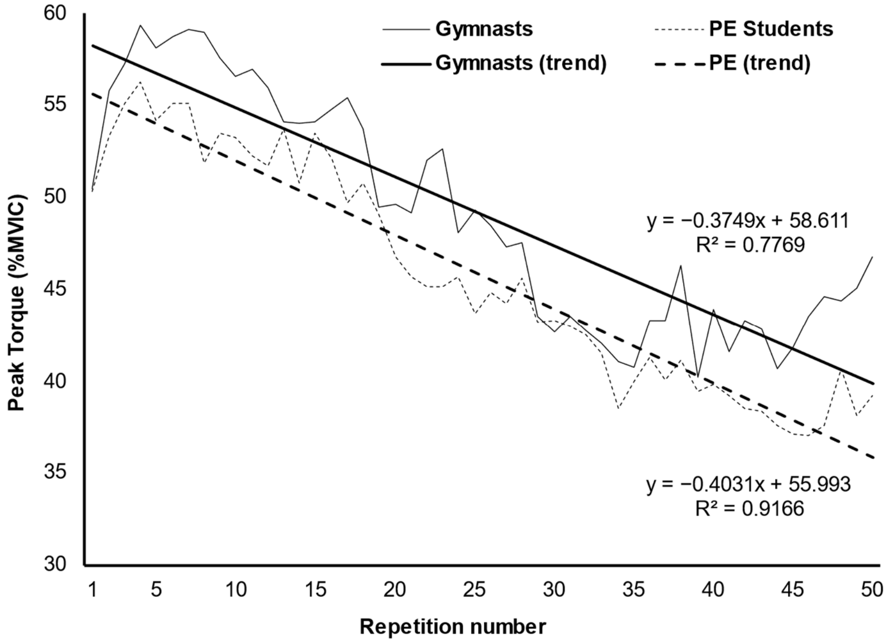

Figure S5. Exploratory linear regression analysis of non-dominant shoulder isokinetic concentric internal rotation peak torque (expressed as %MVIC at 0°, thereby accounting for differences in maximal strength between groups) across a 50-repetition fatigue protocol (240°·s^{-1}) in gymnasts (solid line) and physical education students (dashed line). The slope of each regression line represents fatigue rate, while R^2 indicates goodness of fit.

https://doi.org/10.3390/sports14100433

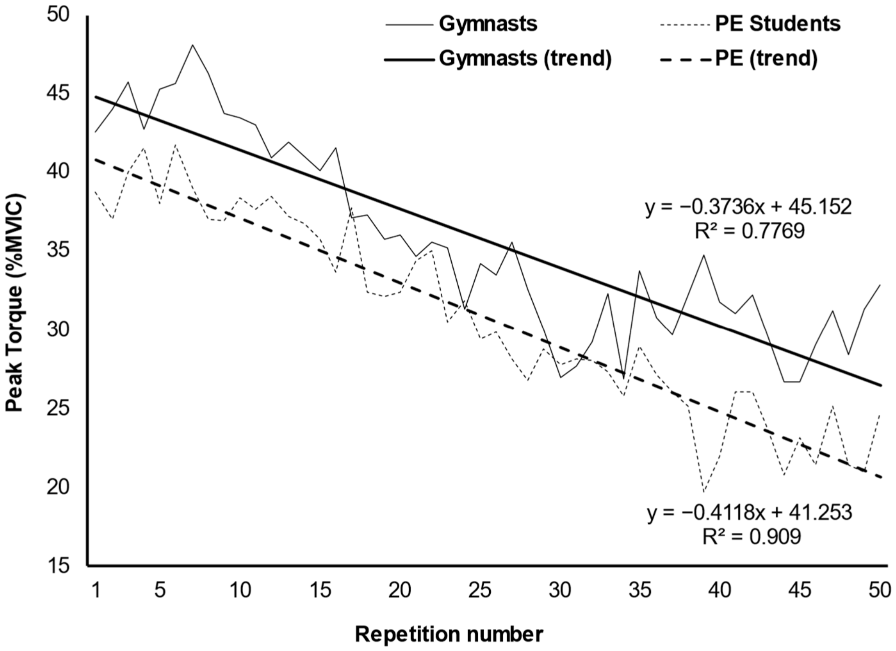

Figure S6. Exploratory linear regression analysis of non-dominant shoulder isokinetic concentric external rotation peak torque (expressed as %MVIC at 0°, thereby accounting for differences in maximal strength between groups) across a 50-repetition fatigue protocol (240°·s^{-1}) in gymnasts (solid line) and physical education students (dashed line). The slope of each regression line represents fatigue rate, while R^2 indicates goodness of fit.

Disclaimer/Publisher's Note: The statements, opinions and data contained in all publications are solely those of the individual author(s) and contributor(s) and not of MDPI and/or the editor(s). MDPI and/or the editor(s) disclaim responsibility for any injury to people or property resulting from any ideas, methods, instructions or products referred to in the content.
