# Supplementary material for: Isometric and Isokinetic Assessment of Shoulder Rotator Muscle Strength, External/Internal Strength Ratios, and Fatigue Response in Competitive Male Gymnasts
Source: Sports (Basel). 2026 Oct 5;14(10):433. doi: 10.3390/sports14100433 (PMC13709291; doi:10.3390/sports14100433)
Supplement: Supplementary file 1 [file sports-14-00433-s001.zip › Supplementary Tables S1_S4_sports-4540623.pdf]

sports

MDPI

Supplementary Material

Exploratory analyses of the remaining strength and ratio conditions—including assessments of the non-dominant shoulder and evaluations at 180°·s⁻¹—and secondary fatigue measures. Exploratory analyses are presented for completeness and hypothesis generation. Statistical estimates and p-values are reported for transparency but were not used to support confirmatory between-group conclusions. Interpretation of exploratory outcomes focused primarily on the magnitude, direction, and precision of observed effects.

Table S1. Exploratory body mass-normalized peak torque (PT) values (M ± SE) before and after the fatigue protocol and mixed ANOVA results for the non-dominant shoulder.

	Gymnasts Pre	PE Pre	Gymnasts Post	PE Post									
Variable	*M* ± *SE*	*M* ± *SE*	*M* ± *SE*	*M* ± *SE*	Group *F*	*p*	η*p*²	Time *F*	*p*	η*p*²	Interaction *F*	*p*	η*p*²
Isom IR 0°	155 ± 5	111 ± 5	148 ± 6	91 ± 6	46.02	< 0.001	0.66	30.82	< 0.001	0.56	10.62	0.003	0.31
Isom ER 0°	58 ± 3	41 ± 3	48 ± 3	29 ± 3	21.52	< 0.001	0.47	22.85	< 0.001	0.49	0.31	0.583	0.01
Con IR 60°·s⁻¹	132 ± 5	87 ± 5	127 ± 5	84 ± 5	43.41	< 0.001	0.64	2.66	0.116	0.10	0.02	0.885	0.00
Con ER 60°·s⁻¹	44 ± 3	33 ± 3	44 ± 3	30 ± 3	10.47	0.004	0.30	2.95	0.099	0.11	1.84	0.188	0.07
Ecc IR 60°·s⁻¹	149 ± 6	99 ± 6	155 ± 7	101 ± 7	36.60	< 0.001	0.60	1.28	0.269	0.05	0.35	0.558	0.01
Ecc ER 60°·s⁻¹	59 ± 3	45 ± 3	63 ± 3	39 ± 3	25.02	< 0.001	0.51	0.66	0.426	0.03	14.85	< 0.001	0.38
Con IR 180°·s-1	116 ± 4	80 ± 4	121 ± 4	77 ± 4	46.53	< 0.001	0.66	0.25	0.623	0.01	5.39	0.029	0.18
Con ER 180°·s-1	34 ± 3	26 ± 3	36 ± 2	22 ± 2	12.81	0.002	0.35	0.33	0.573	0.01	3.51	0.073	0.13
*Con IR 180°·s⁻¹	112 ± 5	82 ± 5	111 ± 5	78 ± 5	25.60	< 0.001	0.52	0.98	0.332	0.04	0.37	0.547	0.02
*Con ER 180°·s⁻¹	39 ± 3	30 ± 2	38 ± 2	28 ± 2	14.49	< 0.001	0.38	0.35	0.561	0.01	0.01	0.906	0.00

Note. All torque values are expressed as Nm·kg⁻¹ × 100. Abbreviations: *M*, mean; *SE*, standard error; *F*, F statistic; *p*, probability value; η*p*², partial eta squared; Isom, isometric contraction; IR, internal rotation; ER, external rotation; Con, concentric contraction; Ecc, eccentric contraction.
*Exploratory concentric contraction at 180°·s⁻¹ for the dominant shoulder.

Table S2. Exploratory conventional and functional external/internal ratios (M ± SE) before and after the fatigue protocol and mixed ANOVA results for the non-dominant shoulder.

	Gymnasts Pre	PE Pre	Gymnasts Post	PE Post									
Variable	*M* ± *SE*	*M* ± *SE*	*M* ± *SE*	*M* ± *SE*	Group *F*	*p*	η*p*²	Time *F*	*p*	η*p*²	Interaction *F*	*p*	η*p*²
Isom ER 0°/Isom IR 0°	38 ± 3	37 ± 3	32 ± 3	34 ± 3	0.01	0.912	.00	8.17	0.009	0.25	0.41	0.527	0.02
Con ER/Con IR 60°·s⁻¹	34 ± 3	39 ± 3	35 ± 2	36 ± 2	0.73	0.401	.03	1.57	0.223	0.06	3.33	0.080	0.12
Ecc ER/Ecc IR 60°·s⁻¹	40 ± 3	47 ± 3	41 ± 2	41 ± 2	1.35	0.257	.05	4.65	0.041	0.16	8.23	0.008	0.26

Ecc ER/Con IR 60°·s^{-1}	46 ± 3	54 ± 3	50 ± 3	48 ± 3	0.54	0.471	.02	0.25	0.625	0.01	11.82	0.002	0.33
Con ER/Con IR 180°·s^{-1}	30 ± 2	32 ± 2	30 ± 2	30 ± 2	0.21	0.653	.01	0.72	0.405	0.03	1.23	0.278	0.05
*Con ER/Con IR 180°·s^{-1}	35 ± 2	37 ± 2	35 ± 1	32 ± 1	0.17	0.680	0.01	7.01	0.014	0.23	3.83	0.062	0.14

Abbreviations: *M*, mean; *SE*, standard error; *F*, F statistic; *p*, probability value; η*p*², partial eta squared; Isom, isometric contraction; IR, internal rotation; ER, external rotation; Con, concentric contraction; Ecc, eccentric contraction; Ecc ER/Con IR, functional ratio.

* Exploratory conventional ratio for the dominant shoulder.

Table S3. Exploratory mixed repeated-measures ANOVA results for normalised peak torque across the 50-repetition fatigue protocol.

Variable	Group *F*	*p*	η*p*²	Block *F*	*p*	η*p*²	Interaction *F*	*p*	η*p*²
IR non-Dom	1.06	0.314	0.04	19.41	< 0.001	0.92	0.80	0.475	0.03
ER non-Dom	1.79	0.193	0.07	30.38	< 0.001	0.95	0.92	0.397	0.04

Note: Greenhouse–Geisser corrected values were used where the assumption of sphericity was violated. Abbreviations: *F*, F statistic; *p*, probability value; η*p*², partial eta squared; Block, 10 consecutive blocks of 5 repetitions; IR non-Dom, internal rotation for the non-dominant side; ER non-Dom, external rotation for the non-dominant side.

Table S4. Exploratory descriptive comparison of fatigue-related indices between groups.

		Group				
**Rotation condition**	**Variable**	**Gymnasts mean ± SD**	**PE students mean ± SD**	**t**	**95%CI**	**Cohen's d**
IR non-Dom	Fatigue index	18.70 ± 19.90	29.53 ± 11.25	−1.71	[−23.92, 2.24]	−0.67
	Absolute drop	11.46 ± 13.25	15.06 ± 5.15	−0.91	[−11.74, 4.54]	−0.36
	Fatigue slope	−0.38 ± 0.28	−0.40 ± 0.16	0.22	[−0.16, 0.20]	0.09
ER non-Dom	Fatigue index	28.57 ± 16.01	39.30 ± 9.17	−1.55	[−25.03, 3.56]	−0.61
	Absolute drop	13.59 ± 9.26	16.61 ± 11.26	−0.75	[−11.38, 5.33]	−0.28
	Fatigue slope	−0.37 ± 0.27	−0.42 ± 0.31	0.50	[−0.18, 0.29]	0.19

Note. Values are presented as Mean ± SD. The *p*-values and 95% CIs were derived from a two-sided independent samples *t*-test. Cohen's d was calculated using the pooled standard deviation; negative values indicate lower values in gymnasts than in PE students. Abbreviations: SD, Standard Deviation; CI, Confidence Interval; IR non-Dom, internal rotation for the non-dominant side; ER non-Dom, external rotation for the non-dominant

Disclaimer/Publisher's Note: The statements, opinions and data contained in all publications are solely those of the individual author(s) and contributor(s) and not of MDPI and/or the editor(s). MDPI and/or the editor(s) disclaim responsibility for any injury to people or property resulting from any ideas, methods, instructions or products referred to in the content.

https://doi.org/10.3390/sports14100433
